# Protein differences between human trapezius and vastus lateralis muscles determined with a proteomic approach

**DOI:** 10.1186/1471-2474-12-181

**Published:** 2011-08-10

**Authors:** Jenny Hadrévi, Fredrik Hellström, Thomas Kieselbach, Christer Malm, Fatima Pedrosa-Domellöf

**Affiliations:** 1 Department of Integrative Medical Biology, Anatomy, Umeå University, S-901 87 Umeå, Sweden; 2 Centre for Musculoskeletal Research, Department of Occupational and Public Health Sciences, University of Gävle, P.O. Box 7629, S-907 12 Umeå, Sweden; 3 Department of Chemistry, Biochemistry, Umeå University, S-901 87 Umeå, Sweden; 4 Department of Surgical and Perioperative Sciences, Sports Medicine, S-901 85 Umeå University, Umeå, Sweden; 5 Winternet, Intendenturvägen 11, S-961 36 Boden, Sweden; 6 Department of Clinical Sciences, Ophthamology, Umeå University, S-901 87 Umeå, Sweden

## Abstract

**Background:**

The trapezius muscle is a neck muscle that is susceptible to chronic pain conditions associated with repetitive tasks, commonly referred to as chronic work-related myalgia, hence making the trapezius a muscle of clinical interest. To provide a basis for further investigations of the proteomic traits of the trapezius muscle in disease, two-dimensional difference gel electrophoresis (2D-DIGE) was performed on the healthy trapezius using vastus lateralis as a reference. To obtain as much information as possible from the vast proteomic data set, both one-way ANOVA, with and without false discovery rate (FDR) correlation, and partial least square projection to latent structures with discriminant analysis **(**PLS-DA) were combined to compare the outcome of the analysis.

**Results:**

The trapezius and vastus lateralis showed significant differences in metabolic, contractile and regulatory proteins, with different results depending on choice of statistical approach and pre-processing technique. Using the standard method, FDR correlated one-way ANOVA, 42 protein spots differed significantly in abundance between the two muscles. Complementary analysis using immunohistochemistry and western blot confirmed the results from the 2D-DIGE analysis.

**Conclusions:**

The proteomic approach used in the present study combining 2D-DIGE and multivariate modelling provided a more comprehensive comparison of the protein profiles of the human trapezius and vastus lateralis muscle, than previously possible to obtain with immunohistochemistry or SDS-PAGE alone. Although 2D-DIGE has inherent limitations it is particularly useful to comprehensively screen for important structural and metabolic proteins, and appears to be a promising tool for future studies of patients suffering from chronic work related myalgia or other muscle diseases.

## Background

The trapezius muscle is a common location for chronic work-related musculoskeletal disorders [1], a condition often referred to as trapezius myalgia [2]. Several models regarding the pathophysiology behind trapezius myalgia have been proposed and were reviewed by Visser & Vaan Dieen [3]. Although the exact mechanisms are unclear, a difference in metabolism between healthy and myalgic muscle has been observed [3]. However, differences in metabolism may reflect the differences in fiber type composition [4-6]. Several studies investigating fiber type composition of myalgic trapezius muscle in comparison to healthy muscle show hypertrophy [7,8] of the slow twitch type 1 fibers. A thorough histochemical characterisation of fiber type composition in ascending, transverse and descending portions of the healthy trapezius muscle was previously presented [9,10]. The most well studied portion of the trapezius muscle is the descending part, which is frequently subjected to loading during shoulder and upper extremity movements [11].

The possible significance of the specific features of the trapezius muscle can be appreciated by comparison to other muscles that are not susceptible to work related muscle pain. The vastus lateralis is a reference muscle of choice because it is a well studied human muscle, routinely used for diagnostic muscle biopsies; and is not a subject to work related myalgia. The vastus lateralis muscle has approximately 50% type 1 fibers [12,13], whereas the descending part of the trapezius muscle has a predominance of type 1 fibers, i.e. approximately 65% [4,9]. The trapezius and vastus lateralis muscles also differs in capillary supply [14,15] and androgen receptor content [16]. A broader analysis of the protein composition of the trapezius muscle can be obtained using a proteomic approach, which to the best of our knowledge has not been presented thus far. The vastus lateralis muscle however, has been the subject of a number of proteomic studies [17-22] comparing different muscle groups [19,23,24], ageing versus young skeletal muscle [20], effects of high altitude [17,18,21] or interval exercise training [22].

In the present study, 2-dimensional difference gel electrophoresis (2D-DIGE) [25] was performed for comparison between the trapezius and vastus lateralis muscles. 2D-DIGE enables a simultaneous quantitative and unbiased analysis of a large number of proteins [19,23,24]. The integration of a pooled internal standard [26] enables a standardization of all gels included in the analysis, lowering the gel to gel differences in the data analysis, which is the main technical variation in human skeletal muscle proteomics [27]. A recent paper by Cairns et al 2009 [28] addressing sample size and technical and biological variation, states the significance of performing in house tests on protocol and sample size. The protocol used in the present study has been validated and tested for repeatability [27].

The vast number of protein spots detected in the 2D-DIGE analysis is evaluated statistically for their significant higher abundance in either muscle. Different statistical approaches can be used on this type of large proteomic data sets, where as the most commonly used statistical method is one-way analysis of variance (ANOVA) (p < 0.05) on individual protein spots. To avoid false positives (type-1 error) due to multiple testing a correction for number of conducted tests should be made. Considered as best practice, false discovery rate (FDR) correlation [29] is applied when approaching proteomic data [30]. In addition, the use of multivariate statistical analysis has the advantage of extracting additional information from the vast data set [31]. In the present study we applied one-way ANOVA statistics with and without FDR correlation and multivariate modelling by partial least square projection to latent structures with discriminant analysis (PLS-DA).

To our knowledge, no previous study has used this explorative approach to elucidate the proteome of the human trapezius muscle. This study compares the physiological characteristics of two muscles with different structure and function, the trapezius and vastus lateralis. A large number of protein spots are detected and analysed for significance with one-way ANOVA. Correlation for false positives (FDR) is applied and compared with results without FDR correlation. In addition, multivariate modelling is preformed in order to visualize and compare the results obtained from the different statistical approaches. The proteins identified are spots that differ significantly in abundance between the trapezius and the vastus lateralis muscles according to the FDR correlated one-way ANOVA. The detected proteins are contractile, metabolic and regulatory. Results will serve as a basis for future investigations of the proteomic traits of the trapezius muscle in disease.

## Methods

### Samples

Samples (5-11 mg) were obtained by surgical biopsy from the vastus lateralis muscle and from the descending portion of the trapezius muscle from five healthy male voluntary donors, 25-28 years old. Both biopsies, from the same donor, were taken at the same occasion and by a single surgeon. All subjects gave their informed consent and the study was approved by the regional ethical committee. The muscle samples were rapidly mounted in Tissue-Tek medium (Miles Laboratories, IN, USA), and frozen in isopropanol chilled in liquid nitrogen and stored at -70°C. Muscle tissue to be used for 2D-DIGE was thoroughly cleared from Tissue-Tek before homogenization.

### Two-Dimensional Difference Gel Electrophoresis (2D-DIGE)

The 2D-DIGE method was primarily presented by Alban et al [32] and further developed with the internal standard method by Unlü et al. [25]. The protocol used herein on human skeletal muscle is validated and tested for its repeatability in our lab [27]. Unless otherwise stated, all chemicals were from GE Healthcare, Uppsala, Sweden, and of proteomic grade quality. The frozen muscle samples were suspended in lysis buffer (9.5 M Urea, 4% (v/w) CHAPS and 30 mM Tris Base) and homogenized using a Grinding Kit. The protein content was quantified with a 2D-Quant Kit. A protein yield of approximately 20% of the total biopsy weight was obtained (approximately 1-2.2 mg protein). Protein homogenate samples, containing 50 μg of protein, were labelled with CyDye minimal dyes, Cy2, Cy3 and Cy5, according to manufacturers' protocol. The internal standard method was used [33] incorporating a pooled internal standard labelled with Cy2. In order to reduce variation in the data-set due to difference in characteristics of the different fluorescent dyes, a dye swap was conducted when labelling all the samples. Trapezius and vastus lateralis samples from a single donor were labelled with Cy3 and Cy5, respectively. In samples from the next donor the labelling was reversed, i.e. trapezius labelled with Cy5 and vastus lateralis with Cy3. The samples from one donor, labelled with one of each dye, were pooled to be separated on the same gel and an equal volume of lysis buffer was added to all labelled protein samples. The analytical gels were analysed in a single replicate approach. IPG-buffer pH 3-11 was added to the homogenates to a final concentration of 0.6% (w/v). DeSteak™ rehydration solution was added to a final volume of 450 μl. The samples were applied onto 24 cm 3-11 Non-Linear (NL) Immobilised pH gradient (IPG) strips and rehydrated in the dark for 16 hours, at room temperature. The first dimension was run using an Amersham Ettan™ IPGphor unit applying 300 V for 900 Vhrs, 600 V for 1800 Vhrs, 1000 V for 3000 Vhrs, 5000 V for 55000 Vhrs. Prior to the second dimension each strip was equilibrated for 10 minutes in equilibration buffer (50 mM 1.5 M Tris HCl pH 8.8, 6 M Urea, 30% (v/v) Glycerol (87%), 2% (w/v) SDS, trace of Bromophenol blue and 0.5% DTT). Samples were then alkylated by further equilibration for 10 minutes in the same buffer, containing 4.5% (w/v) iodoacetamide instead of DTT. The second dimension was conducted by loading the strips onto a 12.5% acrylamide gel using the Ettan DALT six apparatus. Gels were run at 5 W per gel, for 30 minutes, followed by additional 5 hours or until the blue front reached the bottom of the gel, with 17 W per gel of a constant temperature of 15°C and in the dark. Gels were then immediately scanned with a Typhoon™ 9410 scanner, using 488 nm laser and emission filter of 520 BP40 for Cy2 labelled proteins, 532 nm laser and emission filter 580 nm BP30 for Cy3 and 633 nm laser and 670 nm BP30 for Cy5. The image was processed using ImageQuant™ V5.2, before protein abundance was determined using DeCyder™ V6.5.

### Statistical analysis

Statistical evaluation of the protein abundance in the 2D-DIGE analysis was made using the Biological Variation Analysis (BVA) module in DeCyder™ V6.5 and multivariate modelling was performed using SIMCA-P 12 (Umetrics AB, Umeå, Sweden). In all statistical analyses, the log of the standardized abundance has been used. The standardized log abundance was derived from the ratio of each pooled protein group (each gel) being normalized by the internal standard. In the Differential In-gel Analysis (DIA) either all 2447 protein spots or 663 protein spots, when applying the exclude filter with the spot volume limit was set to 200 000 a.u. were included in the BVA. Spots with slope values < 1.0 were considered non-protein spots and not included in the analysis. Differences between proteins were assumed to be significant if spots were present in all gels and one-way ANOVA were significant (p < 0.05) between groups. FDR correlations [29] were applied according to the DeCyder manual. No spot volume limit was set to spots included in the multivariate modelling and all spots were required to be present in all gels. In the multivariate analysis (PCA and PLS-DA) the spot volume ratios were mean centred and scaled for unified variance. PCA was used to detect outliers among the observations (i.e technical problems with gels). In the PLS-DA, protein spots with regression coefficients for which the jack-knifed 95% confidence interval did not included 0 and the Variable of importance (VIP) value exceeded 1 were considered of importance. PLS-regression coefficients have recently been shown to be applicable in selection of variables in -omnic data sets when using PLS-DA [34]. Previously, the combination of VIP-values and PLS-regression coefficients provided the most reliable estimation of relevant variables [35]. In the Western blot analysis, differences in relative protein content between the trapezius and vastus lateralis muscle was considered significant if p < 0.05 using an unpaired students t-test.

### Protein identification by MALDI-TOF mass spectrometry

All significant and FDR-correlated protein spots were selected for further identification with matrix assisted laser desorption ionisation time of flight (MALDI-TOF) mass spectrometry. Protein spots identified as interesting in the non-FDR correlated one-way ANOVA and the PLS-DA modelling were not all identified with MALDI-TOF-MS, due to low protein content in the gel spot and a further insufficient access of biopsy material. Gels to be used for protein identification were run as above and used for both manual and automated excision. Protein spots were excised using Ettan™ Spot Handling Workstation (GE Healthcare), with a ∅ 2.0 mm picker head, at the Swegene Proteomics Resource Centre in Lund, Sweden. Gels to be manually excised were loaded with 450 μg unlabelled protein and stained with Coomassie brilliant blue 450 after 2D separation. Mass spectrometry procedures have been described previously [36]. Database searches were carried out using a Mascot server licensed to Umeå University by Matrix science http://www.matrixscience.com using Swiss-Prot and IPI-human databases. The databases were searched using peptide mass fingerprint spectra and post-source decay MS/MS spectra.

### Morphological analysis

Serial cryostat sections, 8 μm thick, were air-dried and then rehydrated in 0.01 M PBS, immersed in 5% non-immune serum and incubated with primary antibodies for 60 min at 37°C or overnight at 4°C. Biopsy sections were dyed with antibodies at several occasions, at least triplicates were made to verify accuracy. Myotilin was detected with a rabbit polyclonal antibody raised against recombinant N-terminal fragment (residues 1-151) [37], and alpha-crystalline beta [38] and NADH ubiquinone oxireductase 30 kDa subunit (NDUSF antibody 17D95) [39] were identified with mouse monoclonal antibody. The mouse monoclonal antibody A4951 against slow myosin heavy chain was used to easily identify the type 1 fibers, and the monoclonal antibody A474 against fast myosin heavy chain, both antibodies were from Developmental Studies Hybridoma Bank maintained by the University of Iowa, Department of Biology, Iowa city, IA, USA. Visualization of bound antibodies was performed with indirect fluorescence using Alexa 488 and Alexa 568 (Molecular Probes Inc., Eugene, OR, USA). The sections were evaluated in a Nikon eclipse E 800 microscope (Nikon Inc., Melville, NY, USA) and a SPOT RT Color camera (Diagnostic Instruments Inc., Sterling Heights, MI, USA) was used for image acquisition. Digital images were processed using the Adobe Photoshop software (Adobe Systems Inc., Mountain View, CA, USA). The same set of trapezius biopsies used in this study have previously been characterized microscopically and contain 64 ± 6% type 1 fibers, 26 ± 10% type 2A fibers and 9.5 ± 8% type 2AB fibers [40].

### Western blot analysis

Equal amounts of protein from pooled samples of either trapezius or vastus lateralis muscles were loaded on precast 10% SDS-page gels (CBS-scientific, Del Mar, CA, USA) and separated by electrophoresis (DCX-700, CBS-scientific, Del Mar, CA, USA) (running buffer: 25 mM Tris, 250 mM Glycine, 0,1%SDS). The proteins were blotted (transfer buffer: 25 mM Tris, 250 mM Glycine, 20% methanol) on a PVDF membrane (Thermo Fisher Scientific, Waltham, MA, USA) using Bio Rad semi-dry blotting system (Bio Rad Laboratories Ltd, Cambridge, UK). Unspecific binding was blocked by 5% w/v nonfat dry milk in TBST (TBS with 0,1% Tween-20) at 4°C over night. The membrane was incubated for 1 hour in RT with primary antibodies (anti-beta-enolase, GeneTex, GTX 113429S, polyclonal rabbit; at concentration 1:1000, anti-phosphoglycerate mutase 2, GeneTex, GTX109582S, polyclonal rabbit; at concentration 1:1000 and anti-alpha-crystalline beta, Novacastra, NCL-ABCrys-512, monoclonal mouse, at 1:200 concentration) in 5% w/v nonfat dry milk in TBST. The membrane was washed and incubated with either secondary anti-rabbit or anti-mouse alkaline-phosphate-conjugated antibodies in 5% w/v nonfat dry milk in TBST for 1 hour in RT (anti-rabbit/AP, DakoCytomation, D 0487, polyclonal goat at concentration 1:1000 or anti-mouse, GenTex, GTX 27062, polyclonal donkey; at concentration 1:1000). The blots were developed by incubating the membrane with BCIP/NBT (Thermo Fisher Scientific Waltham, MA, USA) until desired staining was achieved. The membranes were photographed and analyzed using ChemiDoc XRS (Bio Rad Laboratories Ltd, Cambridge, UK) with Quantity One software version 4.6.6 (Bio Rad Laboratories Ltd, Cambridge, UK). The relative protein content was calculated based on the detection of pixel volumes of the photographed membranes. Background pixel volume was subtracted from each protein band. All western blot analysis was conducted in, at least, triplicates to verify accuracy.

## Results

**Figure 1:**
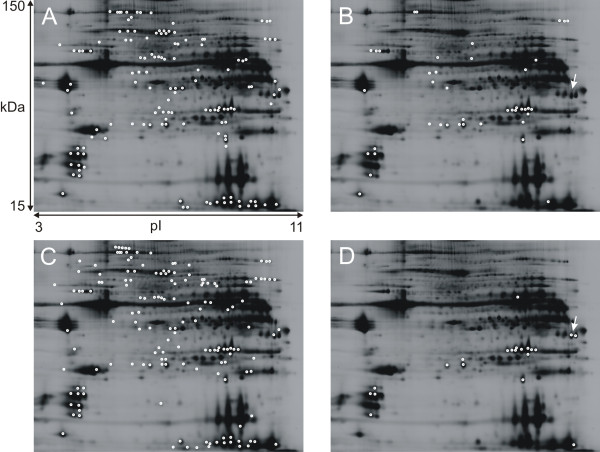
**Protein spots of interest using different statistical and pre-processing techniques**. Trapezius muscle homogenate on 12.5% preparative gel scanned with Typhoon scanner™. pH range 3-11 and molecular weight 15-150 kDa. **1A: **Proteins spots significant according to the one-way ANOVA analysis, not FDR correlated. **1B: **Protein spots significant according to the one way ANOVA and FDR correlated. **1C: **Protein spots of interest in the PLS-DA model: VIP-values higher than 1 and a regression coefficient with a jack-knifed 95% confidence interval not including 0. **1D: **Proteins spots significant according to one-way ANOVA and FDR correlated, when a spot volume exclude filter of 200 000 au is applied. Arrows indicate spots identified as phosphoglycerate mutase 2 (P15259).

A total of 2447 protein spots were detected in the 2D-DIGE gel from vastus lateralis and trapezius muscle extracts. Without applying any spot volume limit in the DIA module, a statistical analysis was made in the DeCyder V6.5 BVA module. Of the 2447 protein spots, 545 spots were present in all gels. No protein spots were exclusive for either muscle. When applying the one-way ANOVA, 140 spots were present in significantly different amounts (p < 0.05) and present in each and every gel (Figure 1A). Forty-two of the 140 protein spots were significant when applying the false discovery rate (FDR) correlation (Figure 1B).

**Figure 2:**
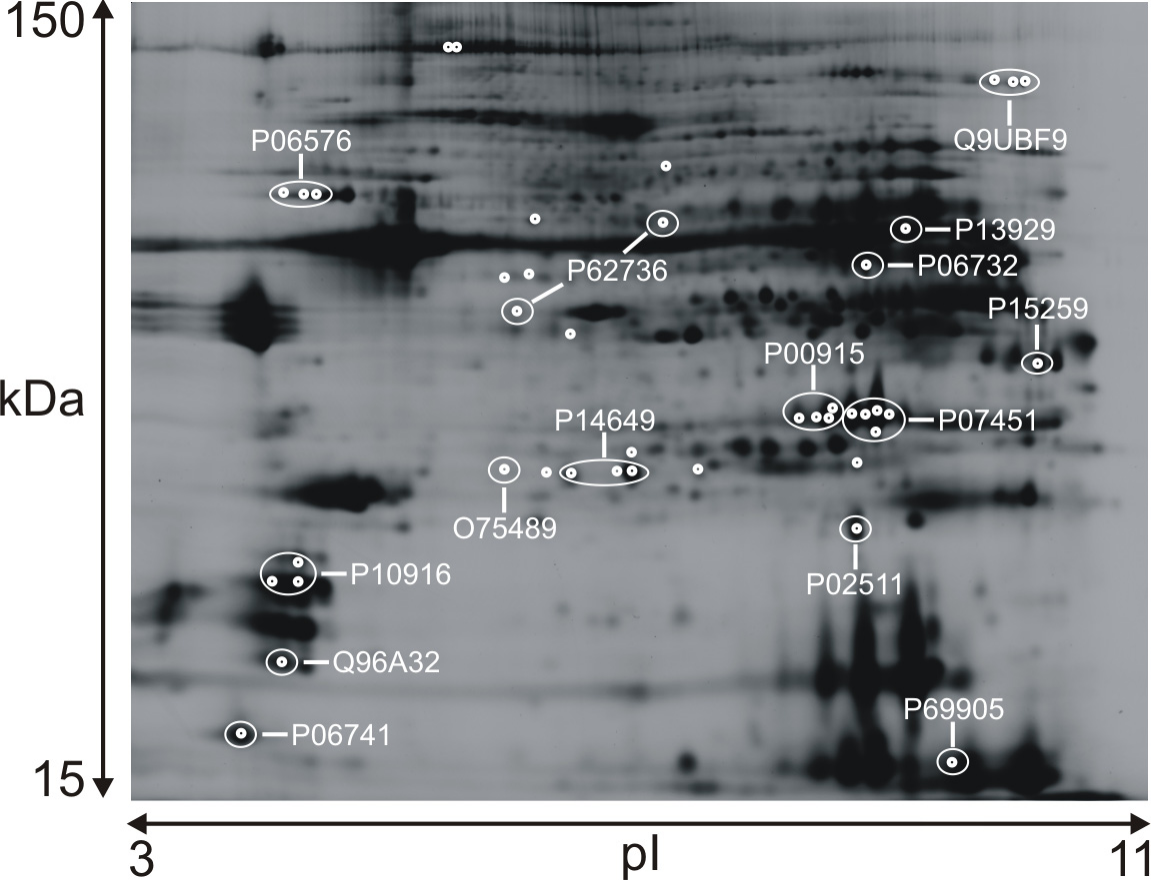
**Preparative 2D-gel with accession numbers for identified proteins**. Typhoon scanner™ image. Cy2 dyed sample from the trapezius muscle within the pH range 3-11 and molecular weight 15-150 kDa. Accession numbers for identified proteins are indicated and listed in Table 1.

Out of 42 significant protein spots 14 different proteins were identified using MALDI-TOF mass spectrometry (Figure 2). The proteins detected related to oxidative metabolism, NADH ubiquinone oxidoreductase 30 kDa subunit (O75489), carbonic anhydrase 1 (P00915) and carbonic anhydrase 3 (P07451), were all more abundant in the trapezius muscle. ATP synthase beta chain (P06576), also related to oxidative metabolism, was more abundant in vastus lateralis compared to trapezius. Creatine kinase M-type (P06732), an enzyme important in anaerobic metabolism, as well as beta enolase (P13929), an enzyme active in glycolysis; were more abundant in the vastus lateralis. Fast myosin light chains (P06741, Q96A32) were significantly more abundant in vastus lateralis whereas slow myosin light chains (P10916, P14649) were more abundant in trapezius. Myotilin (Q9UBF9), smooth muscle actin (P62736) and alpha crystalline beta chain (P02511) were more abundant in the trapezius muscle. Proteins significant at p < 0.01 were NADH ubiquinone oxidoreductase, carbonic anhydrase 1 and 3, alpha crystalline beta, MyLC1A alkali slow and actin aortic smooth muscle (table 1).

**Table 1:** Identified proteins

Swiss prot ID/Protein identifier	Protein name/ description	p-value (ANOVA)	Ratio V/T	Protein mass [Da]	Mascot score	Matched/detected peptides	Sequence coverage
O75489	NADH ubiquinone oxidoreductase	0.00058	-10.39	30242	63	6	33

P00915	Carbonic anhydrase 1	0.0064	-2.21	28778	126	17	60

P02511	Alpha crystalline β	0.0026	-2.09	20146	177	16	69

P06576	ATPsynthase B	0,023	1.57	56525	227	30	58

P06576	ATPsynthase B	0.030	1.52	56525	143	31	50

P06732	Creatine Kinase M-type	0,025	1.35	43302	194	21	55

P06741	MyLC 3 fast alkali	0,030	2.51	16599	143	12	61

P07451	Carbonic anhydrase 3	0.00071	-2.45	29707	81	9	27

P07451	Carbonic anhydrase 3	0.0020	-2.15	29707	146	13	43

P07451	Carbonic anhydrase 3	0.0041	-1.99	29707	162	15	56

P07451	Carbonic anhydrase 3	0.021	-1.83	29707	147	17	72

P10916	MyLC 2, slow regulatory/ventricular	0.015	-2.15	18646	133	14	70

P13929	Beta enolase	0,025	1.69	47168	216	21	58

P14649	MyLC1A alkali slow	0.00058	-10.73	22864	163	7	26

P14649	MyLC1A alkali slow	0.00071	-8.69	22864	71	4	21

P62736	Actin aortic smooth muscle	0.0080	-1.88	42381	70	13	34

P62736	Actin aortic smooth muscle	0.025	-1.80	42381	71	8	22

P69905	Hemoglobulin alpha	0.046	-2.13	15174	63	6	48

Q96A32	MyLC 2 fast regulatory	0.023	1.77	19188	93	13	50

Q9UBF9	Myotilin	0,031	-2.56	55760	169	19	50

Q9UBF9	Myotilin	0,032	-2.17	55760	176	17	41

P15259	Phosphoglycerate mutase 2	0.071	-1.6	28788	126	19	64

Identified proteins significantly different between trapezius and vastus lateralis using FDR correlated one-way ANOVA; and phosphoglycerate mutase 2 (P15259): significantly different only when applying a spot volume exclude filter of 200 000 au. Mascot score values are significant (p < 0.05) when greater than 54.

When setting the protein spot volume limit in the exclusion filter of the DIA module to 200.000 a.u., a total of 663 spots were included in the BVA one-way ANOVA, as compared to 2447 protein spots when no exclusion filter was applied, with 96 protein spots significantly different, and when applying the FDR correlation the number diminished to 26 (Figure 1D). Some protein spots considered statistically significant in the FDR correlated one-way ANOVA when including all 2447 spots (presented in table 1), did not appear as significant according to the FDR correlated one-way ANOVA when using the 200.000 a.u. DIA filter settings, and vice versa, visualised in Figure 1B and 1D. This is due to the spot volume normalization algorithm in DeCyder.

**Figure 3:**
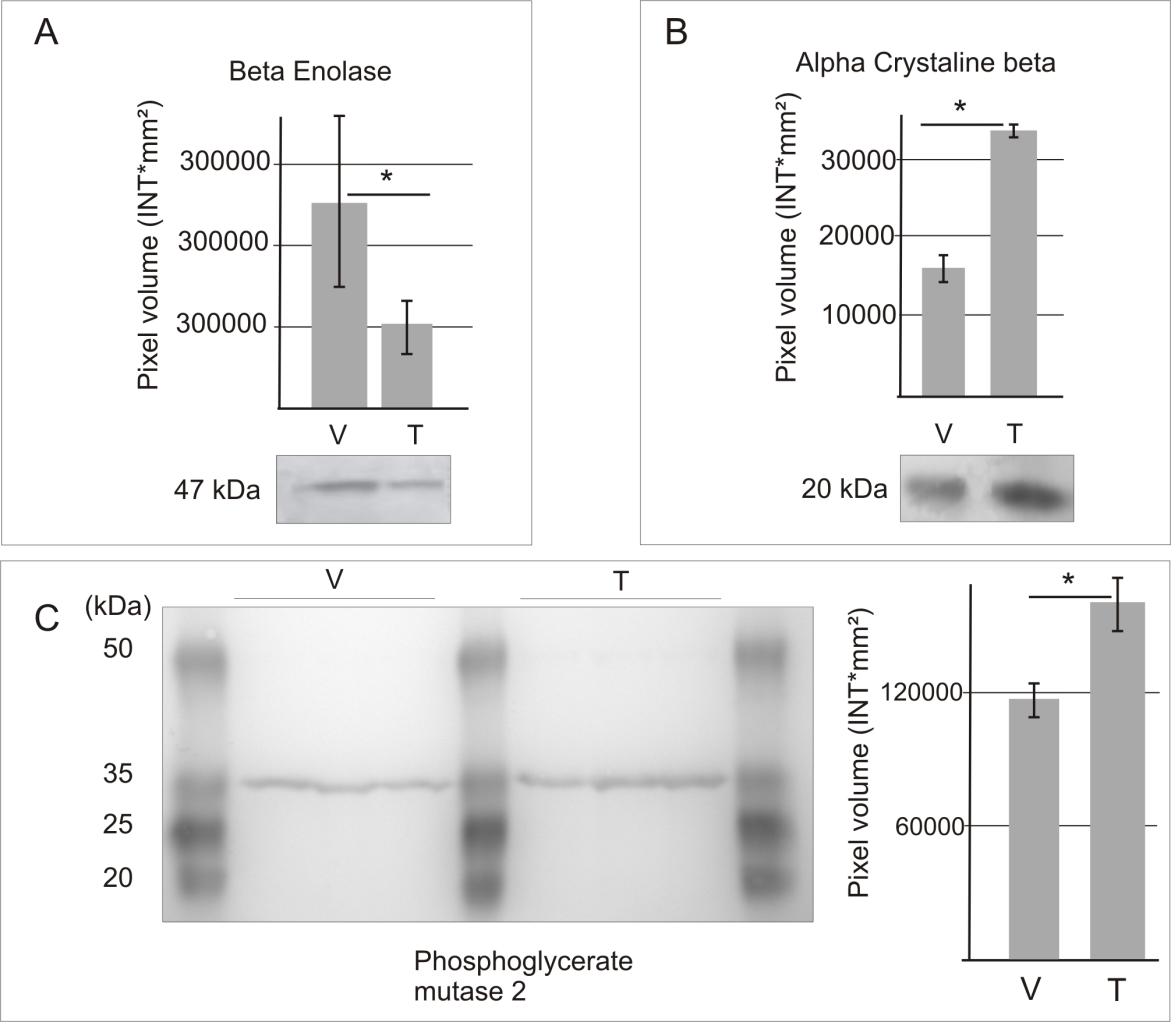
**Western blot results**. Western blots analysis on equal amounts of pooled homogenate from trapezius (T) muscle and vastus lateralis (V) muscle using specific antibodies for: **A**, beta enolase, **B**, alpha crystalline beta and **C**, phosphoglycerate mutase 2. Graphs show mean pixel volumes (intensity *mm2) and error bars show the standard deviation (n = 3). * p < 0.05 (students t-test).

Proteins considered not significant when setting the DIA exclusion filter spot volume limit to 200.000 a.u. where ATP synthase beta chain (P06576), smooth muscle actin (P62736), fast myosin light chain 3 alkali (P06741), myotilin (Q9UBF9) and creatine kinase M-type (P06732). Interestingly, the glycolytic enzyme, phosphoglycerate mutase 2 (P15259) (Figure 1D andtable 1) was not significant in the analysis where all 2447 spots were included. However, it is significant in the analysis where the exclusion filter was applied according to the FDR correlated one-way ANOVA. In the non-FDR correlated one-way ANOVA, where no exclusion filter was applied and all spots are included (presented in Figure 1C), phosphoglycerate mutase 2 was considered significant. When performing a western blot analysis on phosphoglycerate mutase 2, a higher abundance (1.21 fold increased abundance, p < 0.05 students- t-test) in the trapezius muscle was confirmed (Figure 3).

**Figure 4:**
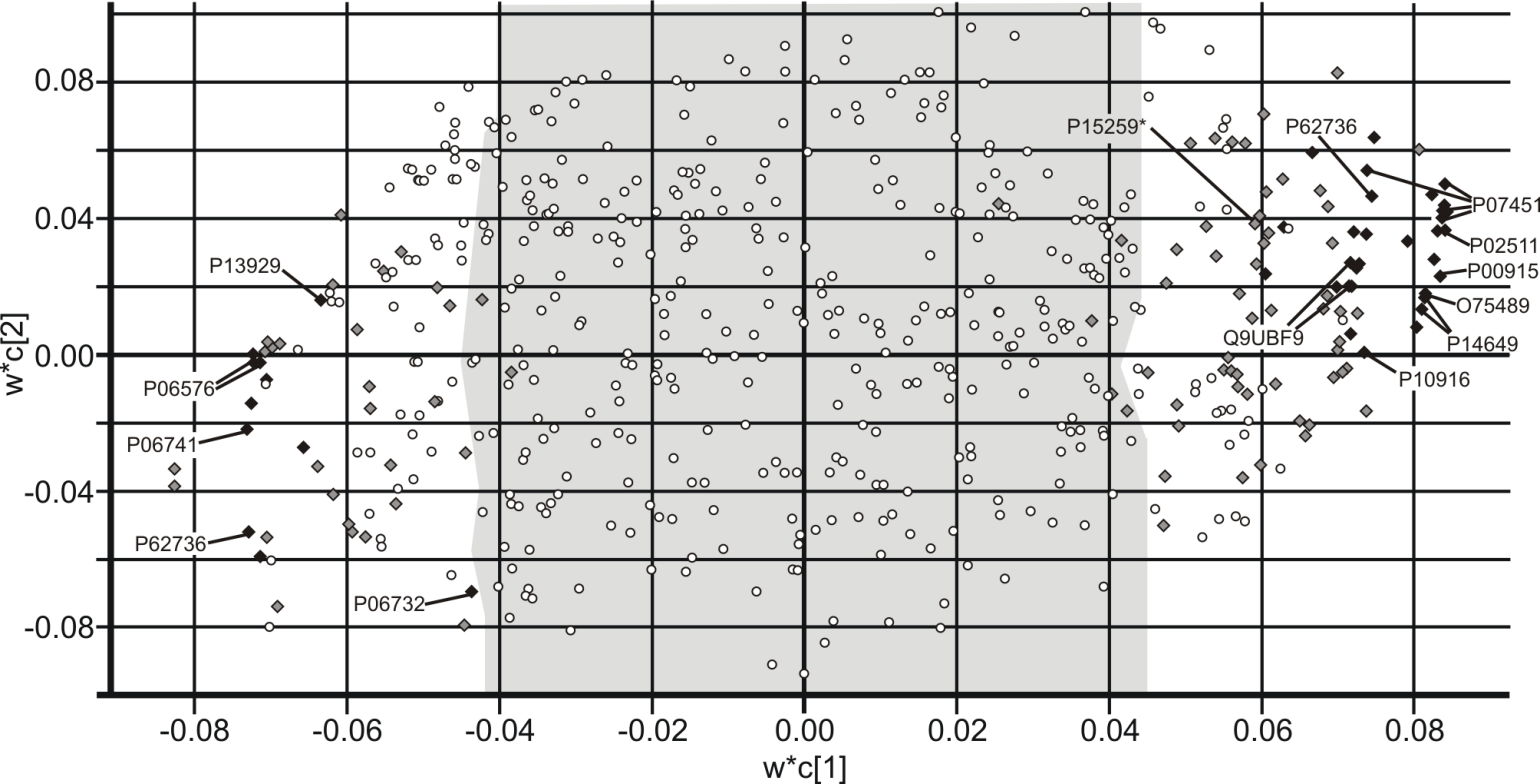
**Protein spot contribution to discrimination between vastus lateralis and trapezius**. PLS-DA weight plot (w*c[1]/w*c[2]) based on 545 protein spots. Situated on the left in the figure are protein spots characterizing the vastus lateralis muscle and on the right the trapezius muscle. Shaded area is proteins spots with variable of importance values (VIP) < 1. Protein spots significant according to the one way ANOVA and FDR correlated (◆). Protein spots significant according to one way ANOVA but not FDR correlated (◆). Protein spots not significant (○). PLS-DA model with two PLS-components ([t1],[t2]) represents 46% of the Y-variance ([t1], 27%, [t2] 19%).

All protein spots, present in 5 out of 5 gels (545 spots) were modelled using PCA and no outliers were found among gels (data not shown). In the PLS-DA analysis (Q^2^_cum _= 0.81 for 2 PLS-components, 27% and 19% explained variance on component 1 and 2, respectively) 164 protein spots (Figure 1C) had a variable of importance (VIP) value higher than 1 and a regression coefficient for which the jack-knifed 95% confidence interval did not included 0 (Figure 4). Spots significant in the above mentioned one-way ANOVA analysis, both with and without FDR-correlation, are visualized in the PLS-DA weights plot (Figure 4), to illustrate the overlap between PLS-DA and one-way ANOVA.

### Validation of differentially expressed proteins

In order to verify the results from the 2D-DIGE, beta enolase (1,67 fold change) alpha crystalline beta chain (2,0 fold change) and phosphoglycerate mutase 2 (1.74 fold change) were selected for western blot analysis and alpha crystalline beta, myotilin and NADH ubiquinone oxireductase were selected for immunohistochemistry. The expression of beta enolase was shown in western blots to be higher in vastus lateralis (1.86 fold change, p < 0.05 students t-test), and alpha crystalline beta chain to be higher in trapezius (2.09 fold change, p < 0.05 students t-test) (Figure 3). The results are in agreement with the proteomics analysis (table 1).

**Figure 5:**
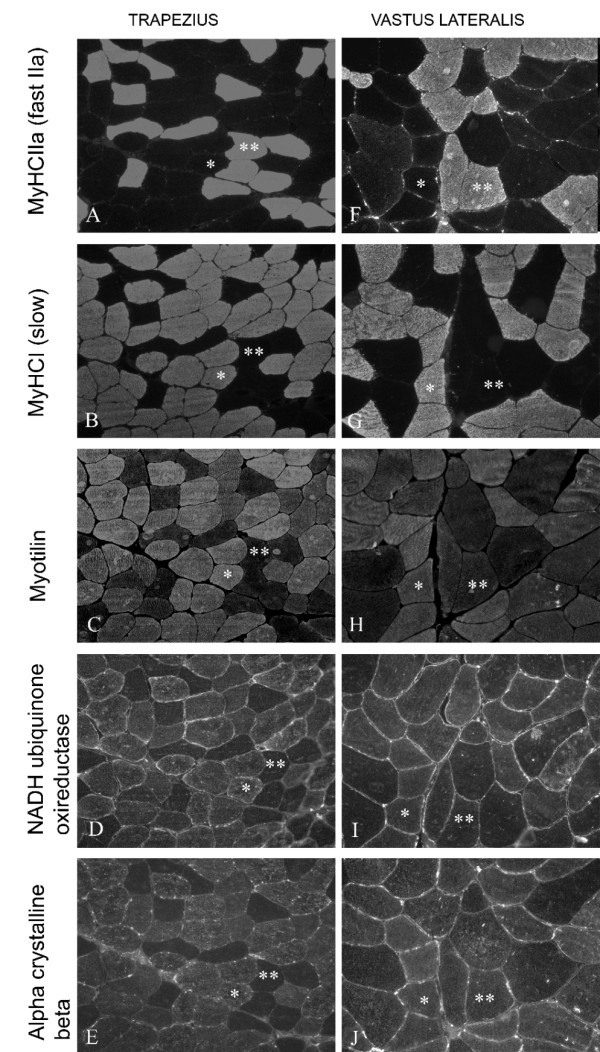
**Serial sections of trapezius and vastus lateralis muscle with fluorescence labelling**. To illustrate the presence of slow and fast muscle fibers, sections were stained with antibodies against MyHCIIa (fast type 2a) (A and F) and MyHCI (slow type 1) (B and G). Adjacent sections were further stained with antibodies against Myotilin (C and H), NADH ubiquinone oxireductase (D and I) and Alpha crystalline beta (E and J); (*type 1 fiber **type 2 fiber). Note the predominance of type 1 fibers in the trapezius muscle and the type 1 fibers higher reactivity to the myotilin, NADH ubiquinone oxireductase and alpha crystalline beta antibodies.

Immunohistochemistry was performed on tissue sections from both the trapezius and the vastus lateralis muscles. Using immunohistochemistry, the exact location of these proteins was determined. Serial tissue sections treated with antibodies against slow and fast myosin heavy chains revealed type 1 and type 2 muscle fibers in a typical salt and pepper pattern (Figure 5). Specific antibodies against alpha crystalline beta, myotilin and NADH ubiquinone oxireductase (Figure 5), representing structural and metabolic proteins, showed higher immunoreactivity in the trapezius muscle, correlating to the predominance of type 1 fibers and thus likely reflecting a relative difference in protein amounts between type 1 and type 2 fibers. Although immunohistochemistry is not a quantitative method, differences in staining intensity within the same tissue section reflect relative differences in protein content.

## Discussion

The protein spots detected to differ significantly in abundance between the trapezius and the vastus lateralis muscles are contractile, metabolic and regulatory proteins that reflect the distinct structural and functional properties of the trapezius muscle. Also, a combination of different statistical methods was applied to obtain a more comprehensive evaluation of the data.

### Major protein findings

The detected differences in content of significant and FDR correlated contractile proteins (e.g. myosin light chains) and metabolic proteins (carbonic anhydrase, NADH ubiquinone oxireductase) are related to fiber type [20]. For example, the results from the 2D-DIGE analysis showed a clear difference in MyLC composition between trapezius and vastus lateralis muscles, with a predominance of slow isoforms in the trapezius muscle (table 1). MyLC2, slow regulatory (P10916) and MyLC1A slow alkali (P14649) were more abundant in the trapezius muscle, compared to the fast isoforms MyLC2 fast regulatory (Q96A32) and MyLC3 fast alkali (P06741) which were more abundant in the vastus lateralis muscle. In healthy adult human muscle the slow isoforms of the alkali and regulatory light chains are expressed in slow type 1 fibers only, whereas the fast alkali isoforms are present in both type 1 slow twitch and type 2 fast twitch fibers [41]. Previous studies have shown that the trapezius has a higher abundance of type 1 fibers compared to vastus lateralis [40,42]. The results from our 2D-DIGE analysis, showing a higher abundance of slow MyLC in trapezius hence are in agreement with earlier results.

Structural proteins may have a more subtle relation to fiber types. In the present study we found that myotilin (Q9UBF9) was more abundant in trapezius compared to vastus lateralis. Myotilin is present in the myofibrillar Z-discs [43]. Type 1 fibers, which are more abundant in trapezius than in vastus lateralis muscle [9,10,42], have broader Z-discs [44] and the trapezius muscle may therefore be expected to exhibit higher abundance of myotilin (Figure 5).

Metabolic proteins also differed between trapezius and vastus lateralis. NADH ubiquinone oxireductase 30 kDa subunit (O75489), one of the subunits in complex 1 of the mammalian mitochondrial electron transport chain and the major superoxide producing component [45], was more abundant in trapezius muscle (Figure 5). ATP synthase beta chain (P06576) is the last complex in the electron transport chain, using protons to build up electron gradient for the phosphorylation of ADP to ATP [46]. It is hence expected that ATP synthase beta chain would be more abundant in the trapezius muscle, in parity with NADH ubiquinone oxireductase 30 kDa subunit, because both proteins are involved in the electron transport chain. Instead there was a higher abundance of ATP synthase beta chain in the vastus lateralis Previous results show a independence in activity between these proteins as both have a suggested reverse action in both consuming and producing protons in the electron transport chain [44,47]. The suggested reverse action of NADH ubiquinone oxidoreductase produces more harmful reactive agents or free radicals, the superoxide anions [48,49], in comparison to the regular activity of the protein.

A preceding proteomic study [19] comparing the vastus lateralis to the deltoideus suggested that two proteins, peroxiredoxin and heat shock protein (HSP) 6β, were adequate markers of fiber type composition and muscle function, having a direct relationship to free radicals homeostasis. In the present analyses peroxiredoxin and HSP6β were not identified among the differentially expressed proteins of trapezius and vastus lateralis, as no significant differences in protein abundance were observed when applying the FDR correlated one-way ANOVA.

### Statistical approaches

In our analysis we detected fourteen proteins involved in contractile, metabolic and regulatory functions of the muscle that differed significantly in abundance between trapezius and vastus lateralis according to the FDR correlated one-way ANOVA (Figure 2, table 1).

This approach, using the one-way ANOVA with FDR correlation is the most frequently used approach in proteomic analysis and is considered to be the correct way to approach proteomic data as it is integrated in the BVA module of the DeCyder program. The FDR correlation should, according to Benjamini and Yekuteli 2001 [50], particularly be used when there is a correlation between variables. Protein spots appearing when using proteomic methods are hence considered to be correlated although proteins are the product of a number of independent translations of genes.

Using multivariate modelling, a general overview of the data is provided [31] proposing a proteomic fingerprint of the muscle. From this fingerprint, proteins with biological relevance can be extracted and further examined with other methods and research approaches. The spots that are significant when using one-way ANOVA and FDR correlation could be considered proteins that are significant as biomarkers, although the biological significance of these proteins must be considered. To obtain as much data as possible, a combination of different univariate and multivariate statistical analysis methods (Figure 1 and 4) is needed in order to ensure that as much information as possible is extracted from the vast data set.

### Methodological considerations

The fact that in the present study not more metabolic enzymes differed significantly between the trapezius and vastus lateralis samples (Figure 2) may be due to methodological issues such as, for example, the choice of buffer solutions, gel density [51] and low abundant proteins not seen due to high abundant proteins. The 2D-DIGE method only allows analysis within a limited pH (3-11) and molecular weight range (15-150 kDa). In order to make a more focused proteomic analysis of the metabolic proteins only, a mitochondrial extraction would be desirable [52]. Though, mitochondrial extractions has limitations as other proteins may contaminate the analysis due to defective purification [53] and hence change the protein to protein relationship. Also, immunohistochemical and immunoblotting approaches have inherent limitations when considering epitope availability.

The present study has shown a number of proteins that differ between trapezius and vastus lateralis muscle thereby revealing the physiological properties of each muscle. Depending on the choice of statistical method and the pre-processing of the gel images, partially different results and conclusions can be drawn from the 2D-DIGE proteomic analysis. When using the stringent FDR correlated one way ANOVA, potential biomarkers for function, disease or structure might be discovered, although the relevant biological significance of proteins found is not considered. To reveal the biological significance of each protein, thorough pathway analyses needs to be performed in order to further elucidate the exact function of each protein and its interaction with other proteins. Careful consideration must be taken regarding the choice of statistical method, depending on what to study and why the analysis is made. As a screening method, 2D-DIGE method is both reliable and sensitive [27] and can reduce the number of proteins targeted for further independent analysis.

## Conclusions

The proteomic approach used in the present study combining 2D-DIGE and PLS-DA as an explorative screening approach provided a comprehensive comparison of the protein profiles of the human trapezius and vastus lateralis muscles, showing significant differences in sarcomeric proteins and proteins related to metabolism. By using the "by the book method", one way ANOVA with FDR correlation, information obtained by the 2D-DIGE method is limited as a number of proteins that may be of interest when using 2D-DIGE is lost. To obtain as much data as possible, a combination of different univariate and multivariate statistical analysis methods is recommended, in order to ensure that all relevant information is extracted from the vast data set. The 2D-DIGE method is particularly useful to comprehensively detect important structural and metabolic proteins, and it is a promising tool for future studies of patients suffering from chronic work related myalgia in the trapezius muscle.

## List of abbreviations

**2D-DIGE**: 2-dimensional difference gel electrophoresis; **ANOVA**: analysis of variance; **ATP**: adenosine tri phosphate; **BVA**: Biological Variation: **DIA**: Differential In-gel Ananlysis; **FDR**: false discovery rate; **HSP**: heat shock protein; **IPG**: Immobilised pH gradient; **MALDI-TOF**: matrix assisted laser desorption ionisation time of flight; **MS**: mass spectrometry; **MyHC**: myosin heavy chain; **MyLC**: myosin light chain; **NADH**: nicotinamide adenine dinucleotide-hydrogen; **PCA**: principal component analysis; **PLS-DA**: partial least square projection to latent structures with discriminant analysis; **VIP**: variable of importance.

## Competing interests

The authors declare that they have no competing interests.

## Authors' contributions

JH participated in design, carried out the 2D-DIGE and MS studies, analysis and interpretation of data; IHC analysis and interpretation; preformed statistical analysis and wrote the manuscript. FH participated in its design, western blot analysis, statistical analysis, interpretation of data and helped to write the manuscript. TK participated in the MS analysis and interpretation of data. CM participated in the 2D-DIGE analysis and interpretation. FPD conceived of the study, participated in its design and helped to write the manuscript. All authors read and approved the final manuscript.

## Pre-publication history

The pre-publication history for this paper can be accessed here:

http://www.biomedcentral.com/1471-2474/12/181/prepub
